# Iron Fortification and Bioavailability of Chickpea (*Cicer arietinum* L.) Seeds and Flour

**DOI:** 10.3390/nu11092240

**Published:** 2019-09-18

**Authors:** Tamanna A. Jahan, Albert Vandenberg, Raymond P. Glahn, Robert T. Tyler, Martin J. T. Reaney, Bunyamin Tar’an

**Affiliations:** 1 Department of Plant Sciences, University of Saskatchewan, Saskatoon, SK S7N 5A8, Canada; taj204@mail.usask.ca (T.A.J.); bert.vandenberg@usask.ca (A.V.); mjr997@mail.usask.ca (M.J.T.R.); 2 Department of Food Science, USDA-ARS, Cornell University, Ithaca, NY 14853, USA; rpg3@cornell.edu; 3 Department of Food and Bioproduct Sciences, University of Saskatchewan, Saskatoon, SK S7N 5A8, Canada; bob.tyler@usask.ca

**Keywords:** chickpea, iron, fortification, NaFeEDTA, hedonic scale, bioavailability

## Abstract

Iron (Fe) deficiency is one of the most common nutritional disorders, and is mainly due to insufficient intake of bioavailable Fe. Chickpea (*Cicer arietinum* L.) was examined as a potential vehicle for Fe fortification. Fortificants (FeSO_4_·7H_2_O (ferrous sulfate hepta-hydrate), FeSO_4_·H_2_O (ferrous sulfate mono-hydrate) and NaFeEDTA (ethylenediaminetetraacetic acid iron (iii) sodium salt)) were applied by a spraying and drying method. At 2000 µg g^−1^ iron fortificant, the fortified split desi seeds (dal), desi flour and kabuli flour supplied 18–19 mg, 16–20 mg and 11–19 mg Fe per 100 g, respectively. The overall consumer acceptability using a nine-point hedonic scale for sensory evaluation demonstrated that NaFeEDTA-fortified cooked chickpea (soup and chapatti) scored the highest among the three fortificants. Lightness (L*), redness (a*) and yellowness (b*) of Fe-fortified products changed over time. However, no organoleptic changes occurred. Fe bioavailability was increased by 5.8–10.5, 15.3–25.0 and 4.8–9.0 ng ferritin mg^−1^ protein for cooked split desi seeds (soup), desi chapatti and kabuli chapatti, respectively, when prepared using Fe-fortified chickpea. Desi chapatti showed significantly higher Fe bioavailability than the other two. The increase in Fe concentration and bioavailability in fortified chickpea products demonstrated that these products could provide a significant proportion of the recommended daily Fe requirement.

## 1. Introduction

Iron (Fe) deficiency, mainly due to low intake of bioavailable Fe, is one of the most common and widespread nutritional deficiencies in the world [1,2,3]. Over three billion people, from both non-industrialized and industrialized countries, suffer from Fe deficiency and Fe deficiency-related anemia, especially women and young children [4]. Fe deficiency causes major health problems such as impaired physical and cognitive development, less capacity for work, increased risk of miscarriage and an increased child mortality rate [1]. Recommended daily allowances (RDAs) for Fe depend on the amount of Fe that is absorbed from the diet and utilized to conduct normal body activities [5]. The daily average Fe requirements at 10% bioavailability for females and males aged 19–50 years are 29.4 mg and 13.7 mg, respectively [6].

Iron fortification of food is a key approach to addressing Fe deficiency [7]. Fe deficiency is prevalent in the population of many developing countries where total calorie intake mainly depends on a grain-based diet. It is estimated that only 2%–5% of the Fe present in legume seeds and cereal grains is bioavailable due to the presence of antinutritional components such as phytate [8]. One strategy to combat iron Fe deficiency is to increase the Fe concentration in the seed or grain through biofortification [9,10]. Fe fortification of the seeds can increase the amount of bioavailable Fe in the diet. Iron fortification of staple food and condiments can act as a primary intervention to supply bioavailable and stable Fe to populations at large [11]. Iron fortification has been scrutinized and implemented in many non-industrialized countries where Fe deficiency is a major public health problem. In India, Fe-fortified whole wheat flour significantly improved body Fe status and reduced Fe deficiency in Fe-depleted children [11]. In Canada, fortification of wheat flour with Fe and some vitamins was first reported in 1944. Thereafter, the childbirth mortality rate decreased from 102 per 1000 live births in 1944 to 61 in 1947 [12,13].

Iron bioavailability is generally considered to be the fraction of dietary Fe that is absorbed and used for normal body functions. For subjects with no Fe stores and who are non-anemic, the ranges of Fe bioavailability for mixed and vegetarian diets were 14%–18% and 5%–12%, respectively [5]. The regulation of Fe absorption by Fe status depends on the solubility and exchangeability of Fe from the food matrix at the intestinal absorption site [14]. To assess Fe bioavailability from foods, in vitro digestion using the Caco-2 cell bioassay is a useful tool for measuring intestinal uptake of Fe [15].

Sensory evaluation provides important and useful information to product developers and food scientists regarding the sensory characteristics of their products [16,17,18]. Iron-fortified foods are used globally to alleviate Fe deficiency; however, the added Fe may cause organoleptic changes in food such as undesirable flavor and color [19]. To determine consumer acceptance of fortified foods, sensory evaluation is required to determine factors that affect food attributes [20]. Lipid oxidation is a process that leads to the formation of several components that cause off-flavors and reduce nutritional quality [21]. Pulse lipids are susceptible to oxidation, which leads to several changes in lipids and other food constituents. Considering Fe fortification in chickpea seeds and flour, two major concerns were rancidity due to oxidation of unsaturated lipids and color changes, as chickpea contains the highest amount of fat (6.04 g 100 g^−1^) among commonly consumed pulses [6,22].

Globally, chickpea is the second most consumed pulse after common bean (*Phaseolus vulgaris* L.) [23]. The average per capita consumption of chickpea worldwide is 1.3 kg year^−1^ or 3.6 g day^−1^. South Asia is the largest consumer of chickpea (averaging 4.25 kg year ^−1^ or 11.6 g day^−1^) followed by the Middle East and Northern Africa (averaging 2.11 kg year ^−1^ or 5.9 g day^−1^) in 2006–2008 [24]. Chickpea is consumed on a daily basis in many developing countries such as India and the surrounding countries [25]. It is consumed in many forms such as whole seeds, dal and besan (flour). Dal (decorticated split cotyledons) is used to make soup, whereas besan is mixed with wheat flour to make chapattis [26]. Chickpea is considered a superb whole food due to its excellent nutritional quality [27]. It is a high quality and inexpensive source of protein for large vegetarian populations in South Asian countries [28,29]. Moreover, chickpea seed is a good source of some essential minerals, including Fe [30]. The average Fe content of chickpea was reported as 3.0–14.3 mg per 100 g of the edible portion [31]. Because chickpea is consumed by large populations in developing countries, it is a potential vehicle for Fe fortification to address Fe deficiency in humans.

To date, no information is available on Fe fortification of chickpea as a potential solution to the Fe deficiency problem. The main objectives of this study were (1) to identify suitable Fe fortificants for the fortification of chickpea seeds and flour; (2) to determine consumer acceptance and preference for Fe-fortified raw and cooked chickpea products through sensory evaluation; and (3) to determine the relative amount of bioavailable Fe in Fe-fortified chickpea products.

## 2. Materials and Methods

### 2.1. Seed Source and Seed Preparation

Two chickpea varieties, CDC 551-1 (average Fe, 41.6 mg kg^−1^) and CDC Frontier (average Fe, 53.2 mg kg^−1^) were used in this study. The former is a “desi” type chickpea with a dark tan seed coat color, and the latter is a “kabuli” type with a beige seed color. The seeds were provided by the Crop Development Centre, University of Saskatchewan. The kabuli chickpea seeds were used with the seed coats. Desi chickpea seeds were dehulled abrasively followed by sieving over 7.5 mm and 11 mm screens. Materials (cotyledons) retained on the 7.5 mm screen were passed through a column blower (Agriculex, model CB-1, Guelph, ON, Canada). The dehulled seeds were collected and stored at room temperature. The moisture content for both varieties was measured by drying samples in a convection oven at 80 °C for 12 h.

### 2.2. Fortificant Preparation and Application Procedures

Three WHO-approved Fe fortificants were used in this study, namely ferrous sulfate heptahydrate (FeSO_4_·7H_2_O), ferrous sulfate monohydrate (FeSO_4_·H_2_O) and sodium Fe EDTA (NaFeEDTA). All the three Fe fortificants were water soluble and relatively cost effective as well as had high relative bioavailability (~100%) compared to ferrous sulfate heptahydrate (FeSO_4_·7H_2_O) [6]. Two types of ferrous salts were tested as they are commonly used in flour fortification. NaFeEDTA, though it is relatively more expensive than the ferrous salts, is one of the best fortificants for food that contains phytic acid [6]. The fortificant solutions were prepared at nine Fe concentrations with 400 µg g^−1^ intervals ranging from 100 to 3200 µg g^−1^. Ten milliliters of each of the Fe fortificant solutions was sprayed onto 100 g of dehulled desi and whole kabuli chickpea seed samples. The samples were then dried at approximately 75 °C by placing them under a 250 watt light bulb with continuous shaking (M49235 Bigger Bill Orbital Shaker, Barnstead Thermolyne, Wilmington, DE, USA) and manual stirring for 15–20 min at 400 rpm. Half of the Fe-fortified desi seed sample and the entire Fe-fortified kabuli seed sample were then ground into flour with a maximum particle size of 106 µm using a cyclone sample mill (UDY Corporation, Fort Collins, CO, USA).

### 2.3. Quality Assessment (Color and Rancidity over Time)

The color of all samples (control and Fe-fortified) from split desi seeds (dal), desi flour and kabuli flour was evaluated using a HunterLab Colorimeter (Hunter Associates Laboratory Inc., Reston, VA, USA). Iron-fortified samples were compared to unfortified controls. The color values (L*, a* and b*) for each sample were recorded at three-month intervals over a one-year period [32].

The fortified desi and kabuli flours along with controls were assessed for rancidity. The samples were stored individually for 6 and 12 months in plastic bags at room temperature (19–22 °C) and a relative humidity of 45%–55%. The moisture contents ranged from 8.3%–9.3%. Five-gram samples were weighed and transferred into test tubes, followed by the addition of 50 mL of methylene chloride to each tube. The flour and methylene chloride mixture was pressed in a syringe and enough oil–solvent mixture collected to recover approximately 1g of oil. The solvent was removed using a rotary evaporator (BUCHI Rotavapoor R-200, Brinkmann Instruments, Inc., Westbury, NY, USA) and the oil was transferred to a 5 mm diameter NMR tube [33]. The samples were analyzed in an NMR spectrometer (Ultrashield 500 MHz/54 mm, Bruker AG, Fallanden, Switzerland) for 1 h to obtain a signal for hydroperoxide, the primary product of lipid oxidation [34].

### 2.4. Meal Preparation

Dehulled desi seeds (500 g), fortified using fortificants containing 2000 µg g^−1^ of FeSO_4_·7H_2_O, FeSO_4_·H_2_O or NaFeEDTA, along with controls (sprayed with similar amount of deionized water with no fortificants), were soaked for 3 h in 3 L of deionized water. Each sample with the same 3 L deionized water that was used for soaking was cooked over medium heat for 40 min. Stainless steel kitchen utensils were used to cook the samples. This resulted in a semi-thick soup, a south Asian traditional chickpea dish called “dal”, to which the following ingredients were added after 20 min of cooking: 20 g of table salt, 10 g of turmeric powder, 30 mL of canola oil and 100 g of chopped onion [35]. Chapattis were prepared using flour from dehulled desi seeds and whole kabuli seeds fortified with 2000 µg g^−1^ of FeSO_4_·7H_2_O, FeSO_4_·H_2_O or NaFeEDTA, along with controls. Chapattis were made from 125 g of the fortified chickpea flour (“besan”) and 125 g of all-purpose refined wheat flour mixed with 2.5 mg of table salt, 38 mL of canola oil and 60 mL of warm water in a stainless-steel bowl. Another 30 g of all-purpose refined wheat flour was used during rolling of the chapattis using a wooden rolling pin and board. The chapattis were then fried on a cooking device of 250 mm diameter and 2100 W power for 3–5 min, followed by cooling at room temperature for approximately 2 h before transferring them into small, foil containers. The samples were kept at −80 °C for 24 h and then freeze-dried using a FreeZone 12 L Console Freeze Dry System (Model 7759040, Labconco, Kansas City, MO, USA) for 72 h followed by storage at room temperature [36].

### 2.5. Iron Concentration Measurement

#### 2.5.1. Uncooked Samples

The Fe concentrations (µg g^−1^) in the split desi seeds (dal) and both desi and kabuli flours, before and after fortification, were quantified by flame atomic absorption spectrometry (F-AAS, Nova 300, Analytik Jena AG, Jena, Germany) at the analytical laboratory at the Department of Plant Sciences, University of Saskatchewan. Before analysis, the samples were digested using a Vulcan digester (Vulcan 84, Questron Technology, Ontario, CA, USA). The digestion was done with two repeats and three technical replications per repeat. The protocols used for digestion and the analysis of Fe concentration were described previously [31,37].

#### 2.5.2. Cooked Samples

The Fe concentrations in the cooked samples (soup and chapattis) were quantified at the USDA-ARS Robert Holley Center for Agriculture and Health (Ithaca, NY, USA) using ICP-AES (iCAP 6500 series, Thermo Jarrell Ash Corp., Franklin, MA, USA) [15]. One-half gram of ground, freeze-dried sample was used for determination of the Fe concentration with three replications [38,39].

### 2.6. Iron Bioavailability Measurement

The bioavailability of Fe in soup and chapatti samples prepared from fortified chickpea (fortificant Fe concentration of 2000 µg g^−1^ of each of FeSO_4_·7H_2_O, FeSO_4_·H_2_O and NaFeEDTA) was assessed at the USDA–ARS Robert Holley Center for Agriculture and Health (Ithaca, NY, USA) using the in vitro digestion/Caco-2 cell model, where ferritin formation is used as an indicator of Fe uptake [15]. Ground, freeze-dried samples (0.5 g) with three replications were used for determination of Fe bioavailability [38,39].

### 2.7. Sensory Evaluation

Sensory evaluation was completed by 40 participants with a 19–60 years age range. Participants who were pregnant or allergic to chickpea were excluded from the evaluation, since these conditions may alter the sense of taste. This evaluation was conducted twice, with a one-month interval, at the Sensory Evaluation Laboratory, Department of Food and Bioproduct Sciences, University of Saskatchewan. Each evaluation was carried out on a single day from 9:00 a.m. to 4:30 p.m. During evaluation, all booths were illuminated equally with white light and each participant entered a separate booth. All participants were recruited from staff and students at the University of Saskatchewan, and were originally from South Asian countries, mostly from Bangladesh, North Eastern India, Sri Lanka, Nepal and Pakistan. The culinary traditions for chickpea consumption in these countries are similar. Consumers tested 12 cooked food samples (soup and chapattis) and visually assessed 12 corresponding uncooked samples (split seeds and flour).

For the screening of cooked (soup and chapattis) and uncooked chickpea samples (split seeds and flour), respondents provided verbal consent and completed consent forms prior to testing. After reading the consent form, participants were asked if they understood the purpose and objectives of the study, procedures for cooked food samples, potential risks by participating in this research, confidentiality of respondents and data and the right to withdraw and follow up, and were informed that any questions from any participants regarding this research could be addressed to the researchers through the Ethical Research Board, University of Saskatchewan. Signed consent forms were collected from the participants for record keeping purposes.

A “sensory evaluation form” was provided to all participants. Participants were requested to evaluate cooked (soup and chapattis) and uncooked samples (split seeds and flour), along with filling in their participant code, age and sex, as well as the date and sample code. Twelve cooked and uncooked samples, along with controls, were presented in small, white plastic containers, each marked with a three-digit number. Each participant was given a spoon, 30 mL of chickpea soup and 60 g of chickpea chapatti, as well as 50 g of split desi seeds and 30 g of flour prepared from cooked and uncooked samples, respectively. Participants were asked to rinse their mouths before and after testing each of the dishes. For the cooked samples, participants evaluated five attributes: Appearance, odor, taste, texture and overall acceptability, whereas for the uncooked samples, attributes included appearance, odor and overall acceptability only. Participants used a nine-point hedonic scale (from 1 = dislike extremely, 5 = neither like nor dislike, to 9 = like extremely). In addition, participants were allowed to record any opinions regarding this study, which were kept for record keeping purposes. Of the initial 45 participants, five did not attend the second session. Therefore, data from only 40 participants were analyzed.

## 3. Statistical Analysis

The Fe concentrations in fortified uncooked (split seed, flour) and cooked (soup, chapattis) chickpea samples and controls were averaged over three replications with two repeats. Values for the Fe bioavailability of cooked chickpea dishes were also averaged over three replications. Sensory data for cooked samples were averaged across two repeats. Descriptive statistics were used to calculate means ± standard deviations (SDs). The PROC GLM of SAS version 9.4 (SAS Institute Inc., Cary, NC, USA) was used to compare means of Fe concentration, Fe bioavailability and sensory data. The correlation between Fe concentration and Fe bioavailability was calculated using Pearson’s correlation coefficient. Statistical significance was assumed at *p* < 0.05.

## 4. Results and Discussion

### 4.1. Iron Concentration

The effects of three food-grade Fe fortificants (FeSO_4_·7H_2_O, FeSO_4_·H_2_O and NaFeEDTA) at nine Fe concentrations ranging from 100–3200 µg g^−1^, with 400 µg g^−1^ intervals on Fe concentration in split desi seeds, desi flour and kabuli flour are presented in Table 1.

Fortification of split desi seeds significantly increased (*p* < 0.001) the Fe concentration relative to the control of split desi seeds sprayed with deionized water. The Fe concentration in unfortified split desi seeds was 43.8 µg g^−1^, whereas the Fe concentration in unfortified desi flour and kabuli flour was 44.8 µg g^−1^ and 54.3 µg g^−1^, respectively. The Fe concentrations in split desi seeds fortified with FeSO_4_·7H_2_O increased in parallel with the Fe concentrations in the fortificant, and ranged from 64.7 to 384.1 µg g^−1^. The Fe concentration in split desi seeds fortified with FeSO_4_·H_2_O and NaFeEDTA ranged from 59.7 to 333.2 µg g^−1^ and from 58.2 to 361.2 µg g^−1^, respectively. The Fe concentration in desi flour and kabuli flour fortified with FeSO_4_·7H_2_O also increased in parallel with the Fe concentrations in the fortificant. The values ranged from 62.4 to 316.5 µg g^−1^ in desi flour and from 80.9 to 373.2 µg g^−1^ in kabuli flour. The Fe concentrations in desi flour fortified with FeSO_4_·H_2_O and NaFeEDTA ranged from 63.7 to 364.5 µg g^−1^ and from 64.1 to 325.2 µg g^−1^, respectively, and the Fe concentrations in kabuli flour fortified with FeSO_4_·H_2_O and NaFeEDTA ranged from 62.7 to 278.5 µg g^−1^ and from 66.1 to 237.6 µg g^−1^, respectively.

A recent study on dehulled lentil using the same three food-grade Fe fortificants (FeSO_4_·7H_2_O, FeSO_4_·H_2_O and NaFeEDTA) also demonstrated the Fe concentrations in the product increased following the Fe concentrations in fortificants [40]. The main purpose of the current study was to increase the Fe content of chickpea seeds and flour that would be absorbable in the gastrointestinal tract. If the addition of inorganic Fe to chickpea seeds or flour does not detract from the end product quality and the added iron is largely absorbable, Fe fortification would provide a low-cost solution to alleviate Fe deficiency problem in countries where chickpea is a main part of the diet. The previous study of Fe fortification in whole wheat flour demonstrated that flour fortification with NaFeEDTA improved body Fe stores and reduced Fe deficiency in children [38]. Similarly, NaFeEDTA was the most effective Fe fortificant for lentil [40]. Our findings are consistent with these reports.

### 4.2. Iron Bioavailability

**Figure 1:**
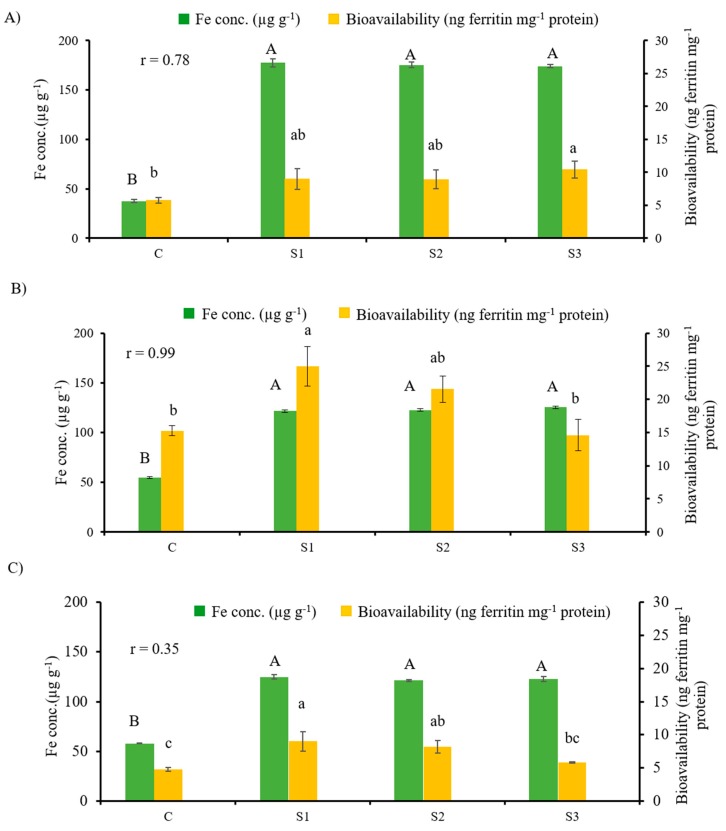
Iron concentration (µg g^−1^) and bioavailability (ng ferritin mg^−1^ protein) of (**A**) cooked split desi seeds (soup) (*n* = 4; C = soup control, S1 = soup fortified with FeSO_4_·7H_2_O, S2 = soup fortified with FeSO_4_·H_2_O, and S3 = soup fortified with NaFeEDTA); (**B**) chapatti prepared from desi flour (*n* = 4, C = desi chapatti control, S1 = desi chapatti fortified with FeSO_4_·7H_2_O, S2 = desi chapatti fortified with FeSO_4_·H_2_O, and S3 = desi chapatti fortified with NaFeEDTA); and (**C**) chapatti prepared from kabuli flour (*n* = 4, C = kabuli chapatti control, S1 = kabuli chapatti fortified with FeSO_4_·7H_2_O, S2 = kabuli chapatti fortified with FeSO_4_·H_2_O, and S3 = kabuli chapatti fortified with NaFeEDTA). Iron bioavailability was analyzed using the in vitro Caco-2 cell model. Upper case and lower case letters represent the mean comparisons of Fe concentrations and Fe bioavailability, respectively, across all the treatments. Bars (mean ± SEM) with the same letters are not significantly different (*p* > 0.05) among the fortificants for that variable.

The iron concentration (µg g^−1^) and bioavailability (ng ferritin mg^−1^ protein) for three cooked chickpea samples (cooked split desi seeds and chapattis prepared from desi flour or kabuli flour) fortified with 2000 µg g^−1^ Fe concentration from FeSO_4_·7H_2_O, FeSO_4_·H_2_O and NaFeEDTA fortificants are shown in Figure 1.

The in vitro Caco-2 cell culture bioassay was used to assess Fe bioavailability. This bioassay procedure calculates Fe bioavailability by measuring cellular ferritin concentration, which is the storage form of Fe. Ferritin is used as a surrogate for bioavailable Fe [5,15,37,39]. Fe concentrations and bioavailability for cooked split desi seeds (soup) are shown in Figure 1A. The effect of Fe fortification on the Fe concentration of cooked split desi seed (soup) was highly significant (*p* < 0.0001). Moreover, for cooked split desi seeds (soup), the relationship between Fe concentration and Fe bioavailability was highly significant, with a correlation coefficient of 0.78 (*p* < 0.05) (Figure 1A). The choice of fortificant had no effect on either the Fe content or the Fe bioavailability of cooked split desi seeds. It has been reported that the absorption of Fe using NaFeEDTA as a fortificant is two to three times higher than with ferrous sulfate in food vehicles with a high phytate content [6,38,40,41,42]. Apparently, cooked split desi seeds (soup) did not contain sufficient phytate to affect the absorption of Fe from the sulfate salts.

Fe concentrations and bioavailability for chapattis prepared from desi flour treated with different Fe fortificants are shown in Figure 1B. The effect of Fe fortification on the Fe concentration of desi chapattis was highly significant (*p* < 0.0001). The relationship between Fe concentration and Fe bioavailability was also highly significant, with a correlation coefficient of 0.99 (*p* < 0.05) (Figure 1B). As was the case for cooked split desi seeds (soup), the choice of fortificant had no effect on the Fe content of desi chapattis. Fe bioavailability did not exhibit a significant difference between FeSO_4_·7H_2_O and FeSO_4_·H_2_O, or between FeSO_4_·H_2_O and NaFeEDTA. Fe bioavailability was greater with FeSO_4_·7H_2_O than with NaFeEDTA *(p* < 0.0001). Since the chapattis were prepared using flour from dehulled desi seeds and refined wheat flour, the anticipated superior absorption of Fe from NaFeEDTA was not observed. The phytate content of refined wheat flour is 2–4 mg g^−1^, compared to 6–10 mg g^−1^ in whole wheat flour [43]. Current findings were consistent with the results of other studies on dehulled lentil and common bean that showed seed coat removal improved Fe bioavailability [37,44].

Iron concentrations and bioavailability for chapattis prepared from fortified kabuli flour are shown in Figure 1C. The effect of Fe fortification on the Fe content of kabuli chapattis was highly significant (*p* < 0.0001). The choice of fortificant had no effect on either the Fe content or the Fe bioavailability of kabuli chapattis. Chapattis prepared from kabuli flour exhibited a moderate correlation between Fe concentration and Fe bioavailability (*r* = 0.35, *p* > 0.05). Chapattis prepared from kabuli flour included the seed coat. Research has shown that colored chickpea seeds (desi) contain more polyphenolic compounds than cream or beige colored seeds (kabuli). However, light colored seed is not completely free of bioactive substances [45]. Furthermore, cooking methods such as baking, frying and roasting retain more polyphenolic compounds compared to soaking and cooking [46]. In the current study, we found that the chapattis prepared from kabuli flour (which included the seed coat) exhibited lower Fe absorption (Figure 1C). This finding was attributed to both seed coat composition and the cooking method (frying).

The relationship between Fe concentration and Fe bioavailability has been reported in previous studies, where an elevated Fe concentration in lentil increased Fe bioavailability [39,47]. Our findings with chickpea provided further confirmation of these reports.

### 4.3. Food Matrix

The effects of three different food matrices (cooked split desi seeds (soup), desi chapatti and kabuli chapatti) and three food-grade Fe fortificants (FeSO_4_·7H_2_O, FeSO_4_·H_2_O and NaFeEDTA) on Fe bioavailability are presented in Supplementary Table S1. The food matrices had a significant effect (*p* < 0.05) on Fe bioavailability. In contrast, the effect of the fortificant was not significant.

The interaction effects between food matrices (cooked split desi seeds (soup), desi chapatti and kabuli chapatti)) and fortificants (FeSO_4_·7H_2_O, FeSO_4_·H_2_O and NaFeEDTA) on Fe bioavailability are presented in Table 2.

Among the three food matrices, the highest Fe bioavailability was observed for chapatti prepared from desi flour (Table 2). The soup of cooked split desi seeds was prepared with spices such as onion, garlic and turmeric that may contain phytic acid and polyphenolic compounds. This may explain the lower Fe bioavailability for cooked desi seeds (soup) than for desi chapatti. This finding agrees with the results of a study that showed Fe bioavailability decreased if the cooked product included spices that contained antinutritional factors such as phytic acid, tannins or other polyphenolic compounds that reduce Fe absorption, digestibility and bioavailability [40]. Since the desi chapatti was made from flour without seed coats, Fe bioavailability was higher than for the kabuli chapatti that included the seed coat. This finding is in agreement with reports on dehulled lentil and common bean where seed coat removal improved Fe bioavailability [37,44].

Across the three food matrices, the choice of fortificant (FeSO_4_·7H_2_O, FeSO_4_·H_2_O or NaFeEDTA) had no effect on Fe bioavailability. Apparently, none the food matrices contained sufficient phytate for the reported superiority of NaFeEDTA as a fortificant to be observed [6].

### 4.4. Quality Assessment (Color and Rancidity over Time)

Color is an important parameter by which consumers judge pulse quality prior to purchase. The color of pulse grain and flour can be affected by the storage period and storage conditions. The effects of nine concentrations of three Fe fortificants (FeSO_4_·7H_2_O, FeSO_4_·H_2_O and NaFeEDTA) on split desi seed, desi flour and kabuli flour included color changes, as reflected in L*, a* and b* values over four time intervals (Supplementary Figure S1). Lightness (L*) of split desi seeds decreased with increasing fortificant concentration, and redness (a*) and yellowness (b*) decreased with time and concentration (Supplementary Figure S1A–C). Similar results were also found for desi flour and kabuli flour (Supplementary Figure S1D–I). These results are similar to those reported in the previous studies for lentil where L*, a* and b* decreased with a longer storage time [40]. Product color affects the commercial value of pulses. A previous study with field pea showed that higher L* and b* values and lower a* values resulted in higher consumer acceptability, which contrasts with our results [48]. Photographs (Supplementary Figures S2–S4) of Fe-fortified split desi seeds, dehulled desi flour and whole kabuli flour at four different storage times show that color changed with time.

Another parameter that determines pulse quality is chemical characteristics. The chemical composition of pulses varies due to their genetic make-up, and storage contributes to changes in their chemical composition [49]. According to the USDA, chickpea contains up to 6% of fat, which is the highest among commonly consumed pulses [22]. A rancidity test was performed to observe organoleptic changes in fortified and unfortified desi and kabuli chickpea flours after six and twelve months of storage under laboratory conditions. The test was done using NMR spectroscopy. Supplementary Figure S5 shows hydroperoxide signals in the range of 10.1–11.0 ppm in the oil of desi and kabuli chickpea controls and fortified flours after twelve months of storage. The rancidity of fat in fortified chickpea flour was compared to that of flax, which showed a hydroperoxide signal in the range of 10.1–11.0 ppm. The oil of Fe-fortified desi and kabuli flours and unfortified controls did not show a hydroperoxide signal in the range of 10.1–11.0 ppm (Supplementary Figure S5). The minimal to no rancidity within the samples could be the result of the laboratory-based storage conditions. More rancidity studies of fortified chickpea under warm and humid conditions are required to determine the potential for off-flavor development and color changes.

### 4.5. Sensory Evaluation

**Figure 2:**
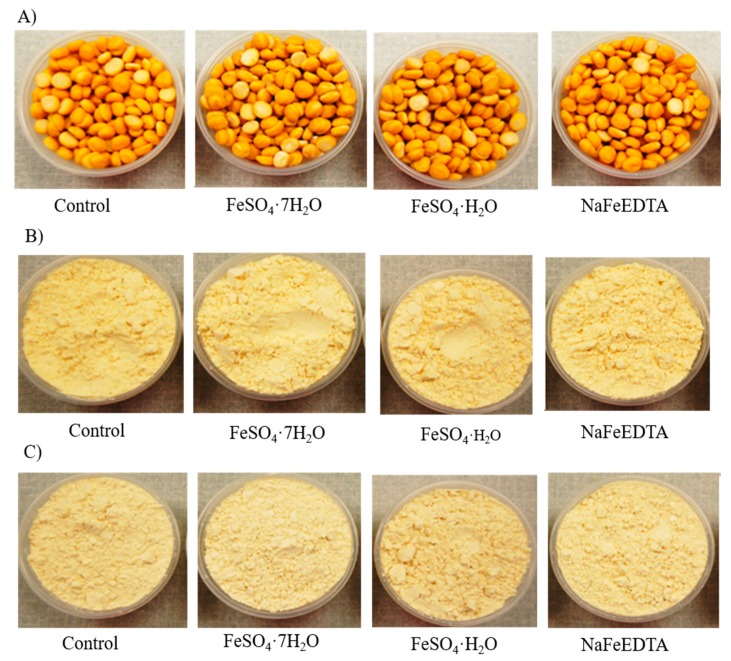
Images of unfortified (control) and Fe-fortified (**A**) uncooked split desi seeds, (**B**) uncooked desi flour and (**C**) uncooked kabuli flour fortified with fortificant solutions containing a 2000 µg g^−1^ of Fe and prepared from three different Fe salts (FeSO_4_·7H_2_O, FeSO_4_·H_2_O and NaFeEDTA).

Sensory attributes are the most expository factors for understanding consumers’ level of acceptance of food [50]. To determine the most suitable fortificant based on consumer preference, sensory evaluation was performed using a nine-point hedonic scale. Participants from South Asian countries assessed three attributes of uncooked samples and five attributes of cooked samples. Figure 2 shows images of the uncooked chickpea products used for sensory evaluation.

Data on the appearance, odor and overall acceptability of the three uncooked products (split desi seed, desi flour and kabuli flour) are presented in Table 3.

The uncooked samples were fortified with three Fe compounds (FeSO_4_·7H_2_O, FeSO_4_·H_2_O and NaFeEDTA). For all three attributes, the control and the FeSO_4_·H_2_O-fortified sample had the lowest and highest scores, respectively, for split desi seed, desi flour and kabuli flour. The mean values for uncooked split desi seeds were 6.9 for appearance, 6.7 for odor and 6.8 for overall acceptability. The mean scores for NaFeEDTA-fortified samples were higher than those of FeSO_4_·7H_2_O- and FeSO_4_·H_2_O-fortified samples for all attributes. The mean scores for Fe-fortified uncooked split desi seeds were significantly lower (*p* < 0.05) than those of the control for all three attributes. For uncooked desi flour, mean scores were 7.5 for appearance, 7.0 for odor and 7.4 for overall acceptability. For the three attributes, the highest scores were for the control and NaFeEDTA-fortified samples. The lowest scores were obtained for FeSO_4_·H_2_O-fortified samples. However, there was no significant variation for any of the attributes across all fortificants, except for uncooked split seeds and overall acceptability in desi flour. For uncooked kabuli flour, mean scores were 6.7 for appearance, 6.5 for odor and 6.6 for overall acceptability. As for uncooked desi flour, NaFeEDTA-fortified samples scored higher than or equal to the control for all attributes. There was no significant variation for the control, NaFeEDTA-, FeSO_4_·7H_2_O- and FeSO_4_·H_2_O-fortified samples for all three sensory attributes. Among the three samples, the mean value for chapattis prepared from desi flour was higher than for cooked split desi seeds and chapatti prepared from kabuli flour for all five sensory attributes, except for taste in cooked split desi seeds. The reason behind this finding is that in sensory evaluation, Fe-fortified and unfortified cooked products from desi flour could not be differentiated from the control due to seed coat, cooking product and method.

Results for five sensory attributes (appearance, odor, taste, texture and overall acceptability) for three cooked products (cooked split desi seeds, chapatti prepared from desi flour and chapatti prepared from kabuli flour) fortified using a fortificant Fe concentration of 2000 µg g^−1^ (FeSO_4_·7H_2_O, FeSO_4_·H_2_O and NaFeEDTA) are presented in Table 4.

**Figure 3:**
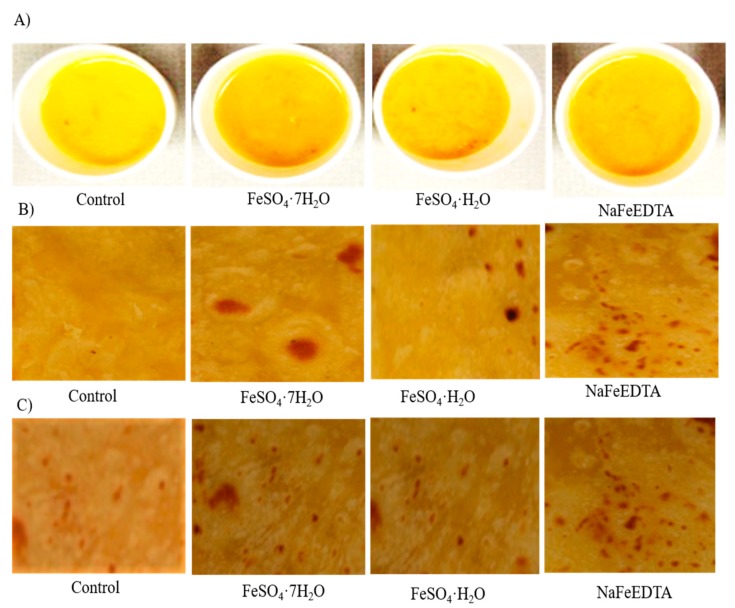
Images of the chickpea products used for sensory evaluation. Images of unfortified (control) and Fe-fortified (**A**) soup prepared from split desi seed, (**B**) chapattis prepared from desi flour and (**C**) chapattis prepared from kabuli flour with 2000 µg g^−1^ of Fe fortificant solution prepared from three different Fe salts: FeSO_4_·7H_2_O, FeSO_4_·H_2_O and NaFeEDTA.

For cooked split desi seeds (soup), the maximum average score was observed for unfortified samples, followed by NaFeEDTA- and FeSO_4_·7H_2_O-fortified samples for all five attributes. The lowest score was obtained for the FeSO_4_·H_2_O-fortified product. Mean scores for cooked split desi seeds were 7.1 for appearance, 6.9 for odor, 7.2 for taste, 6.9 for texture and 7.0 for overall acceptability. For all five attributes, the mean values for Fe-fortified cooked split desi seeds were significantly lower (*p* < 0.05) than for the control. The mean scores for NaFeEDTA-fortified samples were higher than for FeSO_4_·7H_2_O- and FeSO_4_·H_2_O-fortified samples for all attributes. These findings are similar to those for NaFeEDTA where fortified cooked samples scored higher than did FeSO_4_·7H_2_O- and FeSO_4_·H_2_O-fortified samples. For chapattis prepared from desi flour, the mean scores were 7.4 for appearance, 7.2 for odor, 7.1 for taste, 7.0 for texture and 7.1 for overall acceptability. For chapattis prepared from kabuli flour, the mean scores were 7.0 for appearance, 6.8 for odor, 6.9 for taste, 6.9 for texture and 6.9 for overall acceptability. As for cooked split desi seeds, chapattis prepared from desi flour and kabuli flour exhibited the highest scores for the control and the lowest scores for FeSO_4_·H_2_O-fortified samples. However, in contrast to cooked split desi seeds, FeSO_4_·7H_2_O-fortified desi chapattis and kabuli chapattis exhibited scores higher than or similar to those of NaFeEDTA-fortified desi chapattis. As for cooked split desi seeds, the average scores for Fe-fortified desi chapattis were significantly lower (*p* < 0.05) than those of the control for all five attributes. The attributes of Fe-fortified desi chapattis were not significantly different (*p* > 0.05) from that of the control. Similarly, for kabuli chapattis, Fe-fortified products did not exhibit significant differences (*p* > 0.05) for any of the five attributes. Some studies have found no significant differences between unfortified and fortified rice [51,52]. Our findings for desi chapatti and kabuli chapatti yielded similar results due to the use of the same ingredients in the cooked flour recipe, i.e., half wheat flour and half chickpea flour, as well as the same cooking method, i.e., frying. Using this recipe for chapattis would have reduced any differences between fortified and unfortified products. Figure 3 shows images of the chickpea products used for sensory evaluation.

In the current study, panelists evaluated Fe-fortified cooked products which had been fortified using a fortificant with Fe concentration of 2000 µg g^−1^. It was found that fortificant with this Fe concentration provided approximately 180–190 µg g^−1^, 160–200 µg g^−1^ and 110–190 µg g^−1^ of additional Fe per 100 g in the cooked split desi seeds, desi chapatti and kabuli chapatti, respectively. Therefore, per serving (50 g), Fe-fortified split desi seeds or desi chapatti could supply more than 10 mg of Fe (9–10 mg and 8–10 mg from fortified split desi seeds and chapatti, respectively, plus 2 mg from the base chickpea). A wide range (9–13 mg) in Fe content (6–10 mg from fortified kabuli chapatti plus 3 mg from the base chickpea) was observed for fortified kabuli products. These results indicated that fortified chickpea products could provide a major part of the estimated average requirements (EAR) for Fe. A previous study with lentil showed that Fe fortification using a fortificant Fe concentration of 1600 µg g^−1^ could provide an extra 10 mg of Fe [53].

## 5. Conclusions, Future Research and Potential Application

Chickpea is an economically valuable pulse grown in 59 countries around the world, of which approximately 96% is grown in developing countries. Moreover, chickpea is consumed as a daily staple in many developing countries where the prevalence of Fe deficiency is severe. Chickpea occupies an important role in the protein and mineral micronutrient supply, including Fe, for people who depend on plant-based diets for their total calories, especially in South Asia. However, Fe bioavailability from chickpea is relatively low. Therefore, iron fortification of chickpea is worth considering as a means of providing more Fe to mitigate Fe deficiency. This study demonstrates that it is possible to increase both Fe concentration and the amount of deliverable Fe in foods made from chickpea. In terms of Fe bioavailability, NaFeEDTA did not show any superiority over the two ferrous salts in cooked split desi seeds (soup), which is inconsistent with previous reports and fortification guidelines [6,39], and may reflect a relatively low level of phytate in the flours used. Since three food matrices (cooked split desi seed (soup), desi chapatti and kabuli chapatti) were used in the study, the processing and cooking methods may have affected Fe bioavailability after in vitro digestion. Results from testing of three Fe fortificants showed that NaFeEDTA was the fortificant of choice in terms of consumer preference, color and flavor with cooked split desi seeds (soup), an observation also made in previous studies [53]. However, both desi chapatti and kabuli chapatti had higher Fe bioavailability when fortified with FeSO_4_·7H_2_O, compared to FeSO_4_·H_2_O and NaFeEDTA, and higher consumer preference. A major finding from this study was that Fe bioavailability increased after fortification with a fortificant Fe concentration of 2000 µg g^−1^ for the three Fe fortificants. Approximately 11–12 mg of additional dietary Fe could be provided by a 50 g serving of cooked fortified chickpea products. Therefore, fortified chickpea could provide a major portion of the recommended daily allowance for Fe, which could help to reduce Fe deficiency in target populations.

We now have baseline information for fortifying desi chickpea dal and flour of both desi and kabuli chickpea. To our knowledge, this is the first report on Fe fortification of chickpea seed and flour. This information is important for future commercial Fe fortification of chickpea. Although this laboratory-based study suggested feasibility, effective scaling up of Fe fortification at the industrial level needs to be considered. Given the strong influence of Fe fortification on Fe deficiency, it will be important to effectively market Fe-fortified chickpea dal and flour to consumers. As for other pulses, chickpea dal is processed by washing, soaking and cooking of the dal prior to consumption. This may reduce the nutritional value (Fe content and availability) of fortified split chickpea seed. However, one of the positive aspects of Fe fortification of chickpea flour is that people do not wash flour prior to consumption. Therefore, it is recommended that Fe fortified chickpea products be labelled “ready to cook” and “ready to eat”. Moreover, large-scale human trials in Fe-deficient populations need to be conducted to determine the efficacy of Fe fortification of chickpea in mixed diets. Overall, it is recommended that effective systems for Fe fortification of chickpea dal and flour be established to alleviate Fe deficiency in affected populations.

## Figures and Tables

**Table 1:** Effect of three Fe fortificants at nine Fe concentrations on iron concentration in split desi seeds, desi flour and kabuli flour.

	Concentration	Split Desi Seeds	Desi Flour	Kabuli Flour
	Spray Sol. µg g^−1^	Fe Conc. µg g^−1^	Fe Conc. µg g^−1^	Fe Conc. µg g^−1^
Fortificants	0 (Control)	43.8 ^i^	44.8 ^i^	54.3 ^j^
FeSO_4_·7H_2_O	100	64.7 ^h,i^	62.4 ^h,i^	80.9 ^i^
	400	82.5 ^h^	76.1 ^h^	109.4 ^h^
	800	122.6 ^g^	113.0 ^g^	131 ^g^
	1200	155.8 ^f^	148.1 ^f^	169.4 ^f^
	1600	195.2 ^e^	179.8 ^e^	208.6 ^e^
	2000	238.1 ^d^	208.2 ^d^	240.3 ^d^
	2400	297.5 ^c^	238.2 ^c^	260.4 ^c^
	2800	337.1 ^b^	284.0 ^b^	354.2 ^b^
	3200	384.1 ^a^	316.5 ^a^	373.2 ^a^
	LSD _0.05_	21.5	22.7	18.3
	0 (Control)	43.8 ^i^	44.8 ^i^	54.3 ^i^
FeSO_4_·H_2_O	100	59.7 ^h,i^	63.7 ^h^	62.7 ^h^
	400	71.5 ^h^	78.5 ^h^	90.3 ^g^
	800	118.2 ^g^	115.7 ^g^	92.1 ^g^
	1200	145.7 ^f^	142.2 ^f^	113.2 ^f^
	1600	193.5 ^e^	186.0 ^e^	147.9 ^e^
	2000	222.6 ^d^	222.6 ^d^	178.2 ^d^
	2400	257.3 ^c^	258.4 ^c^	202.3 ^c^
	2800	281.0 ^b^	320.8 ^b^	259.9 ^b^
	3200	333.2 ^a^	364.5 ^a^	278.5 ^a^
	LSD _0.05_	18.5	18.2	6.0
	0 (Control)	43.8 ^i^	44.8 ^j^	54.3 ^h^
NaFeEDTA	100	58.2 ^i^	64.1 ^i^	66.1 ^g^
	400	81.6 ^h^	82.6 ^h^	73.4 ^g^
	800	113.1 ^g^	107.5 ^g^	94.4 ^f^
	1200	160.8 ^f^	144.4 ^f^	119.2 ^e^
	1600	194.0 ^e^	185.6 ^e^	150.6 ^d^
	2000	235.5 ^d^	205.9 ^d^	156.3 ^d^
	2400	266.4 ^c^	232.0 ^c^	200.7 ^c^
	2800	302.3 ^b^	287.3 ^b^	221.0 ^b^
	3200	361.2 ^a^	325.2 ^a^	237.6 ^a^
	LSD _0.05_	15.9	15.1	9.0

Different letters in the same column indicate that the Fe concentrations are significantly different at *p* ≤ 0.05.

**Table 2:** The interaction effects between food matrices (cooked split desi seeds (soup), desi chapatti and kabuli chapatti) and fortificants (FeSO_4_·7H_2_O, FeSO_4_·H_2_O and NaFeEDTA) on Fe bioavailability (ng ferritin mg^−1^ protein).

Food Matrix	Fortificants	Fe Bioavailability (ng Ferritin mg^−1^ Protein)
Cooked split desi seeds (soup)	Control	5.8 ^b^
	FeSO_4_·7H_2_O	9.0 ^a,b^
	FeSO_4_·H_2_O	8.9 ^a,b^
	NaFeEDTA	10.5 ^a^
Desi chapatti	Control	15.3 ^b^
	FeSO_4_·7H_2_O	25.0 ^a^
	FeSO_4_·H_2_O	21.5 ^a,b^
	NaFeEDTA	14.6 ^b^
Kabuli chapatti	Control	4.8 ^c^
	FeSO_4_·7H_2_O	9.0 ^a^
	FeSO_4_·H_2_O	8.2 ^a,b^
	NaFeEDTA	5.8 ^b,c^

Means in the same column with same letters are not significantly different based on LSD tests (*p* > 0.05).

**Table 3:** Hedonic scale responses for three uncooked chickpea samples (split desi seed, desi flour and kabuli flour) fortified with three Fe fortificants (FeSO_4_·7H_2_O, FeSO_4_·H_2_O and NaFeEDTA) along with the control for three sensory attributes: appearance, odor and overall acceptability.

Chickpea Samples		Sensory Attributes		
	Fortificants	Appearance	Odor	Overall Acceptability
Uncooked Split Desi Seeds	Control	7.8 ± 0.1 ^a^	7.3 ± 0.2 ^a^	7.6 ± 0.1 ^a^
	FeSO_4_·7H_2_O	6.6 ± 0.2 ^b^	6.4 ± 0.2 ^c^	6.5 ± 0.2 ^b,c^
	FeSO_4_·H_2_O	6.2 ± 0.2 ^c^	6.2 ± 0.2 ^c^	6.3 ± 0.2 ^c^
	NaFeEDTA	7.0 ± 0.2 ^b^	6.8 ± 0.2 ^b^	6.9 ± 0.2 ^b^
Uncooked Desi Flour	Control	7.7 ± 0.1 ^a^	7.1 ± 0.2 ^a^	7.6 ± 0.1 ^a^
	FeSO_4_·7H_2_O	7.5 ± 0.1 ^a^	6.9 ± 0.2 ^a^	7.3 ± 0.1 ^a^
	FeSO_4_·H_2_O	7.3 ± 0.2 ^a^	6.8 ± 0.1 ^a^	7.1 ± 0.2 ^b^
	NaFeEDTA	7.6 ± 0.1 ^a^	7.1 ± 0.2 ^a^	7.4 ± 0.1 ^a^
Uncooked Kabuli Flour	Control	6.8 ± 0.2 ^a^	6.6 ± 0.2 ^a^	6.6 ± 0.2 ^a^
	FeSO_4_·7H_2_O	6.5 ± 0.2 ^a^	6.5 ± 0.2 ^a^	6.5 ± 0.1 ^a^
	FeSO_4_·H_2_O	6.5 ± 0.2 ^a^	6.2 ± 0.2 ^a^	6.4 ± 0.2 ^a^
	NaFeEDTA	6.8 ± 0.2 ^a^	6.7 ± 0.2 ^a^	6.7 ± 0.2 ^a^

Means in the same column with same letters are not significantly different based on LSD tests (*p* > 0.05).

**Table 4:** Hedonic scale responses for three cooked chickpea samples (soup prepared with split desi seed, chapattis prepared from desi flour and chapattis prepared from kabuli flour) fortified with Fe fortificants (FeSO_4_·7H_2_O, FeSO_4_·H_2_O and NaFeEDTA) at a fortificant Fe concentration of 2000 µg g^−1^.

		Sensory Attributes				
Chickpea Sample	Fortificant	Appearance	Odor	Taste	Texture	Overall Acceptability
Soup	Control	7.8 ± 0.1 ^a^	7.5 ± 0.1 ^a^	7.9 ± 0.1 ^a^	7.5 ± 0.1 ^a^	7.6 ± 0.1 ^a^
	FeSO_4_·7H_2_O	6.9 ± 0.2 ^b^	6.8 ± 0.2 ^b^	6.7 ± 0.2 ^b^	6.5 ± 0.2 ^b^	6.6 ± 0.2 ^b^
	FeSO_4_·H_2_O	6.7 ± 0.2 ^c^	6.5 ± 0.2 ^b^	6.8 ± 0.1 ^b^	6.6 ± 0.1 ^b^	6.6 ± 0.1 ^b^
	NaFeEDTA	7.0 ± 0.1 ^b^	6.9 ± 0.2 ^b^	7.2 ± 0.2 ^b^	6.9 ± 0.2 ^b^	7.0 ± 0.2 ^b^
Desi Chapatti	Control	7.7 ± 0.1 ^a^	7.5 ± 0.1 ^a^	7.4 ± 0.2 ^a^	7.2 ± 0.2 ^a^	7.4 ± 0.2 ^a^
	FeSO_4_·7H_2_O	7.4 ± 0.2 ^b^	7.2 ± 0.2 ^b^	7.3 ± 0.2 ^a^	7.1 ± 0.2 ^a^	7.1 ± 0.2 ^a,b^
	FeSO_4_·H_2_O	7.0 ± 0.1 ^c^	6.7 ± 0.2 ^c^	6.7 ± 0.2 ^b^	6.6 ± 0.2 ^a^	6.7 ± 0.2 ^a^
	NaFeEDTA	7.3 ± 0.2 ^b^	7.2 ± 0.2 ^b^	7.0 ± 0.2 ^a,b^	7.0 ± 0.2 ^a^	7.1 ± 0.2 ^a,b^
Kabuli Chapatti	Control	7.2 ± 0.1 ^a^	6.9 ± 0.2 ^a^	6.9 ± 0.2 ^a^	7.0 ± 0.2 ^a^	7.0 ± 0.2 ^a^
	FeSO_4_·7H_2_O	7.0 ± 0.1 ^a^	6.8 ± 0.2 ^a^	6.8 ± 0.2 ^a^	6.9 ± 0.2 ^a^	6.8 ± 0.2 ^a^
	FeSO_4_·H_2_O	6.9 ± 0.1 ^a^	6.8 ± 0.1 ^a^	6.8 ± 0.2 ^a^	6.7 ± 0.1 ^a^	6.8 ± 0.2 ^a^
	NaFeEDTA	6.9 ± 0.2 ^a^	6.8 ± 0.2 ^a^	7.0 ± 0.2 ^a^	6.8 ± 0.1 ^a^	6.8 ± 0.2 ^a^

Means in the same column with the same letter are not significantly different based on LSD tests (*p* > 0.05).

## Supplementary Materials

The following are available online at https://www.mdpi.com/2072-6643/11/9/2240/s1, Figure S1A–I Represent the effects of FeSO_4_·7H_2_O, FeSO_4_·H_2_O and NaFeEDTA with nine different doses on color space of desi dehulled split seeds, desi flour and kabuli flour samples. Each number shows (a) L* (lightness) score (b) a* (redness) score (c) b* (yellowness) score, and (d) L*, a*, b* score at four different storage time at 2000 µg g^−1^ Fe concentration. Figure S2a–d Images of unfortified (control) and iron-fortified dehulled and split desi seeds with 2000 µg g^−1^ of Fe salt solution prepared from three different Fe salts: FeSO_4_·7H_2_O, NaFeEDTA and FeSO_4_·H_2_O at four different time. Figure S3a–d Images of unfortified (control) and iron-fortified desi flour with 2000 µg g^−1^ of Fe salt solution prepared from three different Fe salts: FeSO_4_·7H_2_O, NaFeEDTA and FeSO_4_.H_2_O at four different time intervals. Figure S4a–d Images of unfortified (control) and iron-fortified kabuli flour with 2000 µg g^−1^ of Fe salt solution prepared from three different Fe salts: FeSO_4_·7H_2_O, NaFeEDTA and FeSO_4_·H_2_O at four different time intervals. Figure S5A representative sample flaxseed oil shows the hydroperoxide signals in the range of 10.1–11.0 ppm. (B–I) After 12 months of storage of desi flour control and desi flour fortified with FeSO_4_·7H_2_O, NaFeEDTA and FeSO_4_·H_2_O, and kabuli flour control and kabuli flour fortified with FeSO_4_·7H_2_O, NaFeEDTA and FeSO_4_·H_2_O with 2000 µg g^−1^, respectively, shows no H_2_O_2_ signals in the range of 10.1–11.0 ppm. Table S1. Analysis of variance of the main effects of three different food matrices (desi seeds, desi flour and kabuli flour) and three food-grade Fe fortificants (FeSO_4_·7H_2_O, FeSO_4_·H_2_O and NaFeEDTA) including control on Fe bioavailability in soup, desi chapattis and kabuli chapattis.
