# Supplementary material for: Iron Fortification and Bioavailability of Chickpea (Cicer arietinum L.) Seeds and Flour
Source: Nutrients. 2019 Sep 18;11(9):2240. doi: 10.3390/nu11092240 (PMC6770251; doi:10.3390/nu11092240)
Supplement: Supplementary file 1 [file nutrients-11-02240-s001.zip › Supplementary Figure S1.docx]

Supplementary Figure S1.


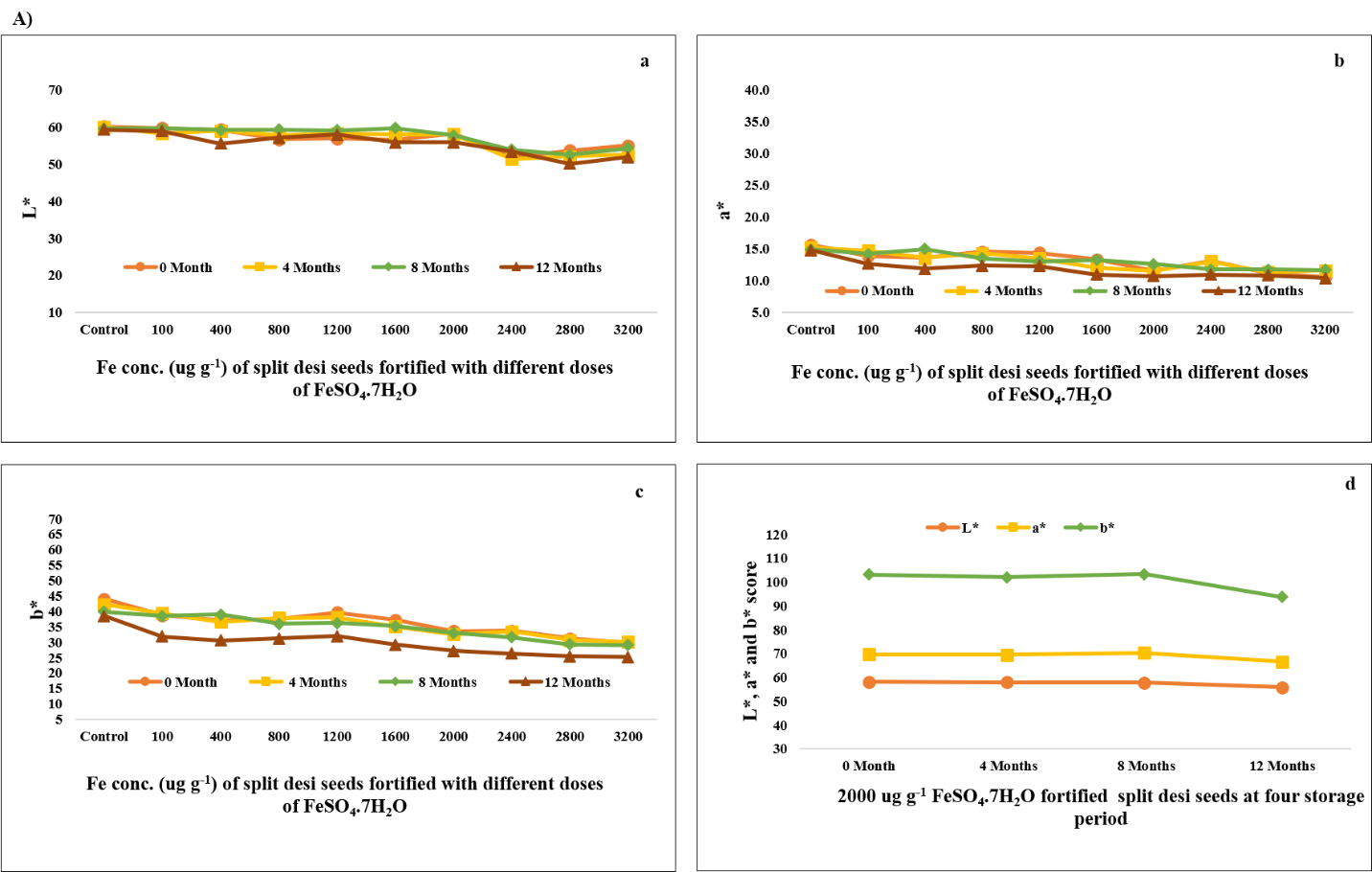


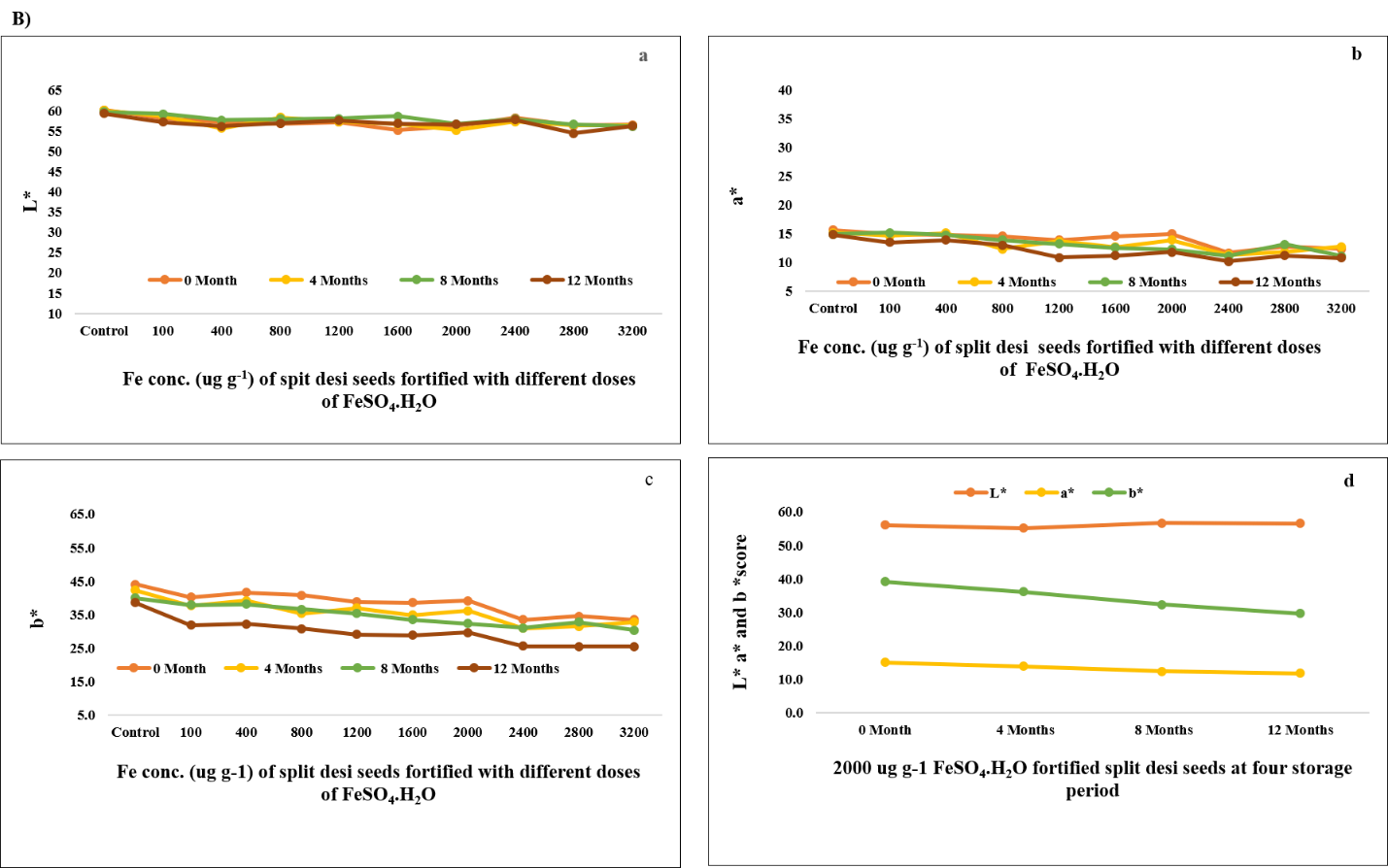


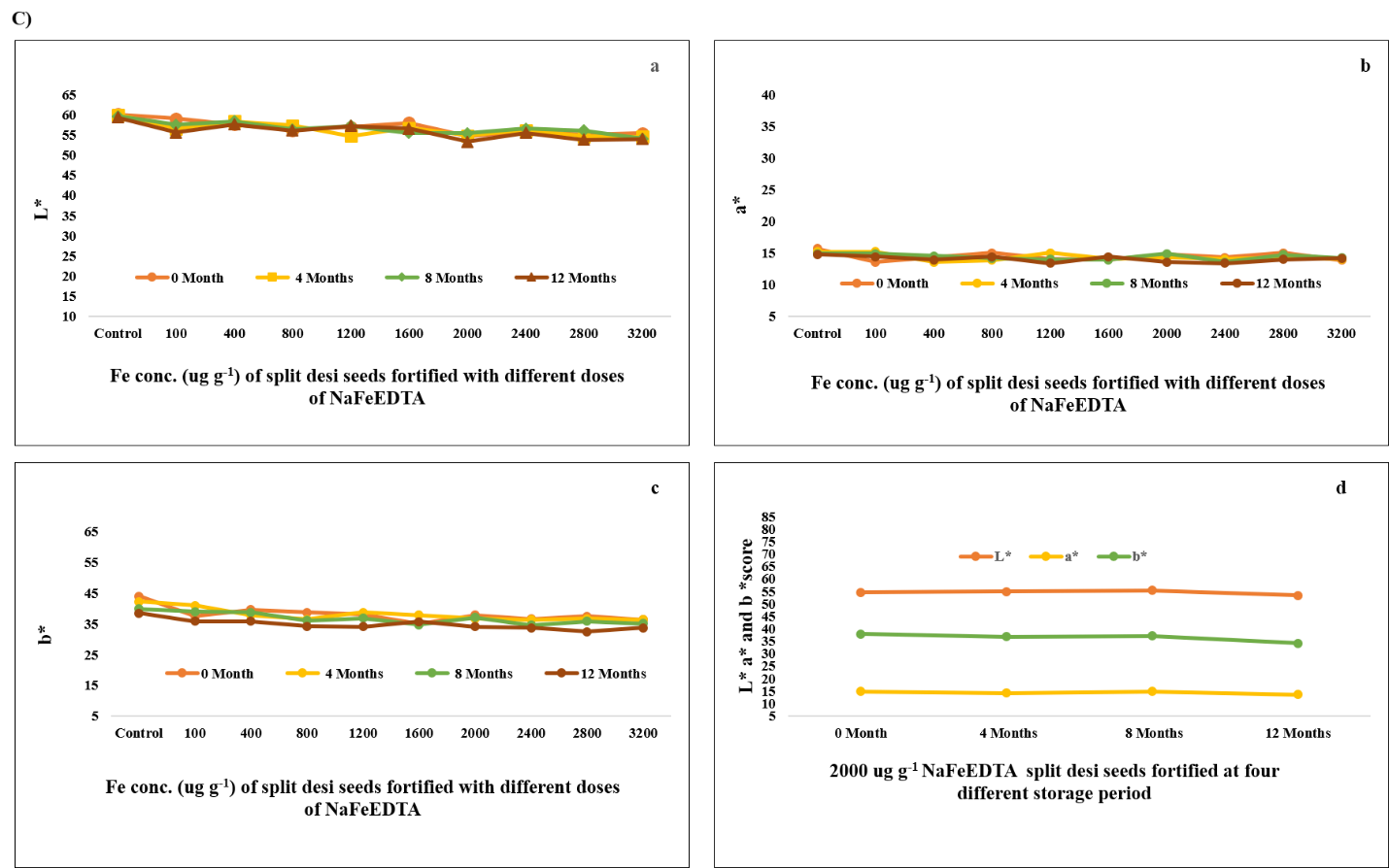


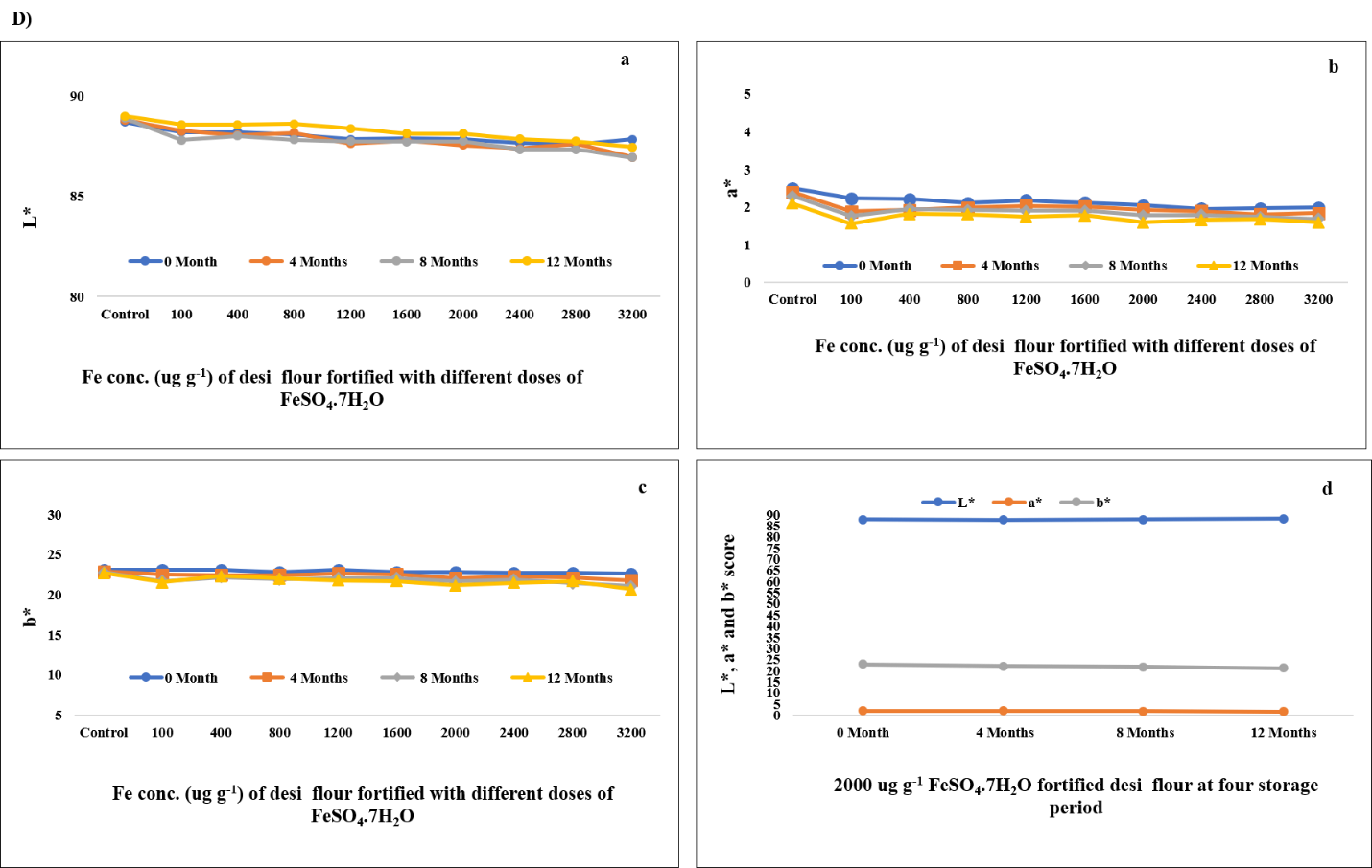


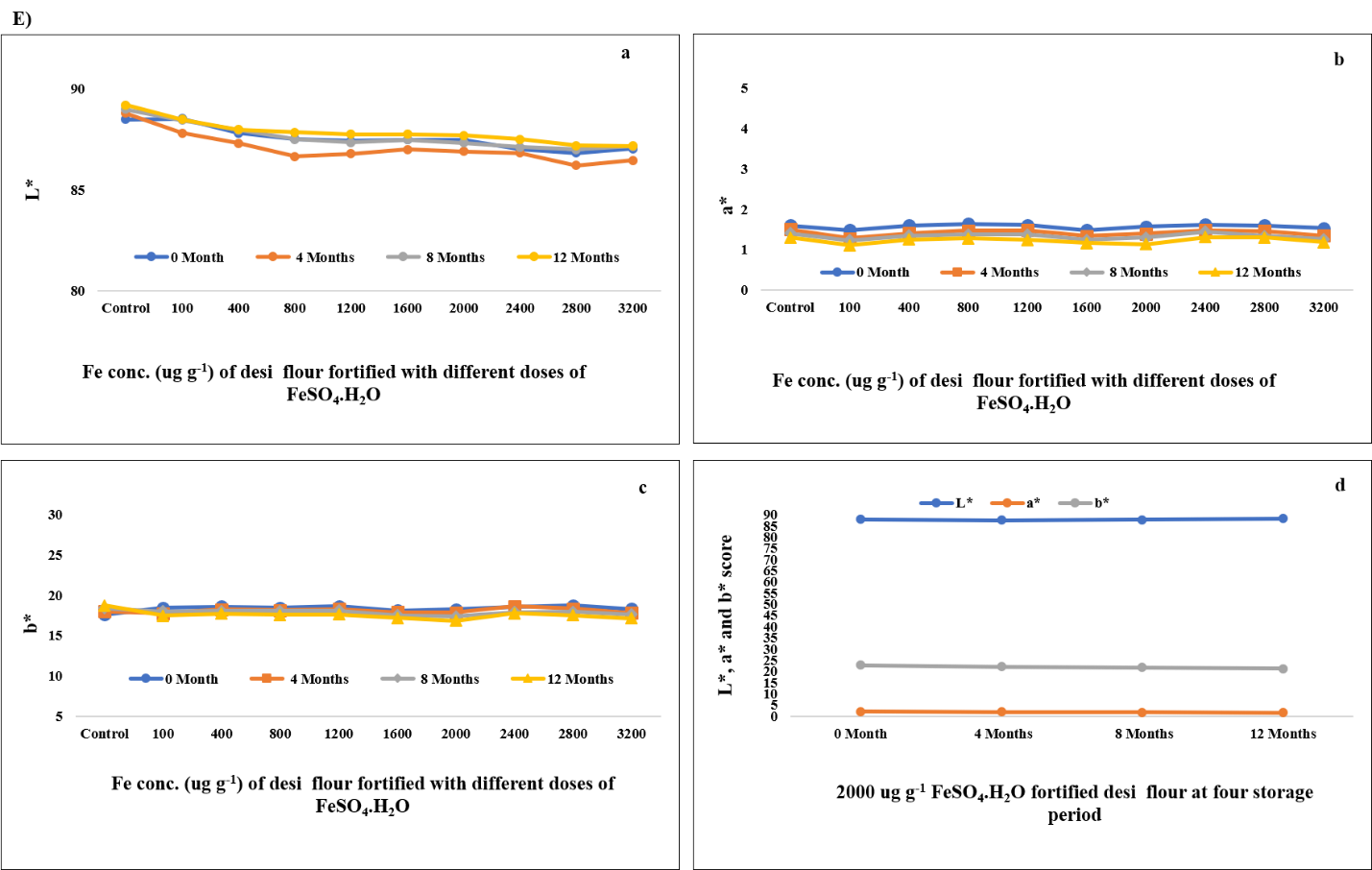


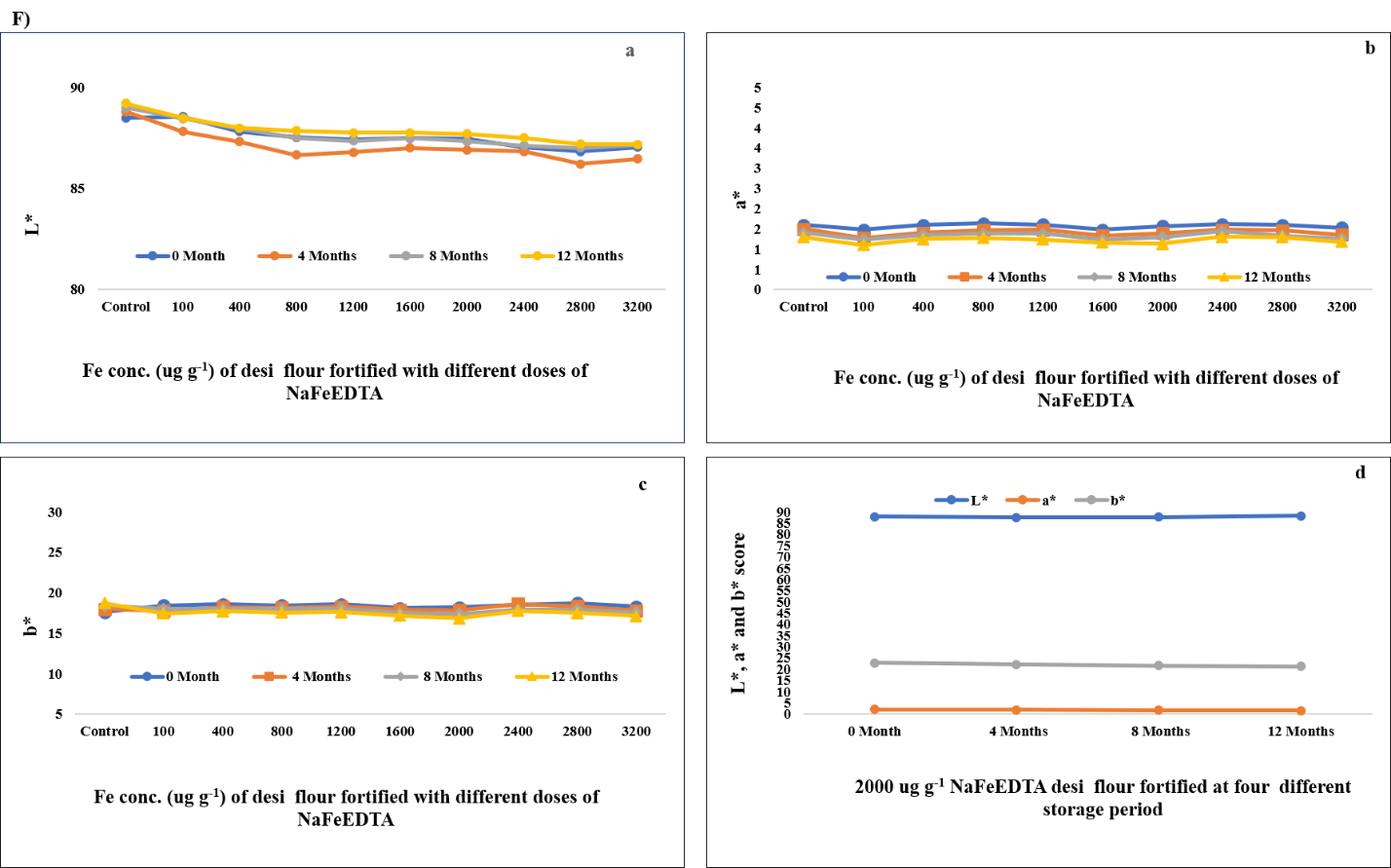


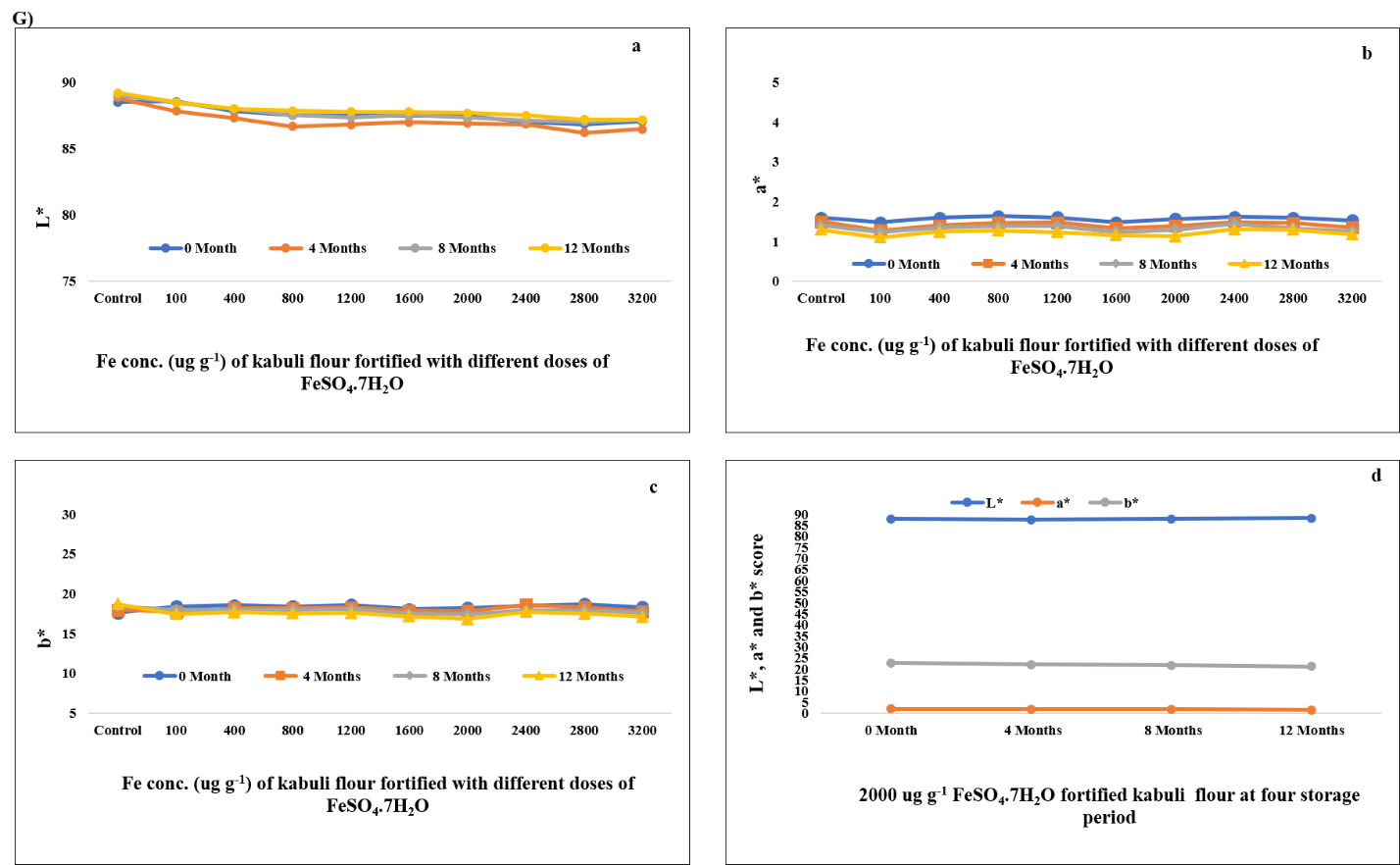


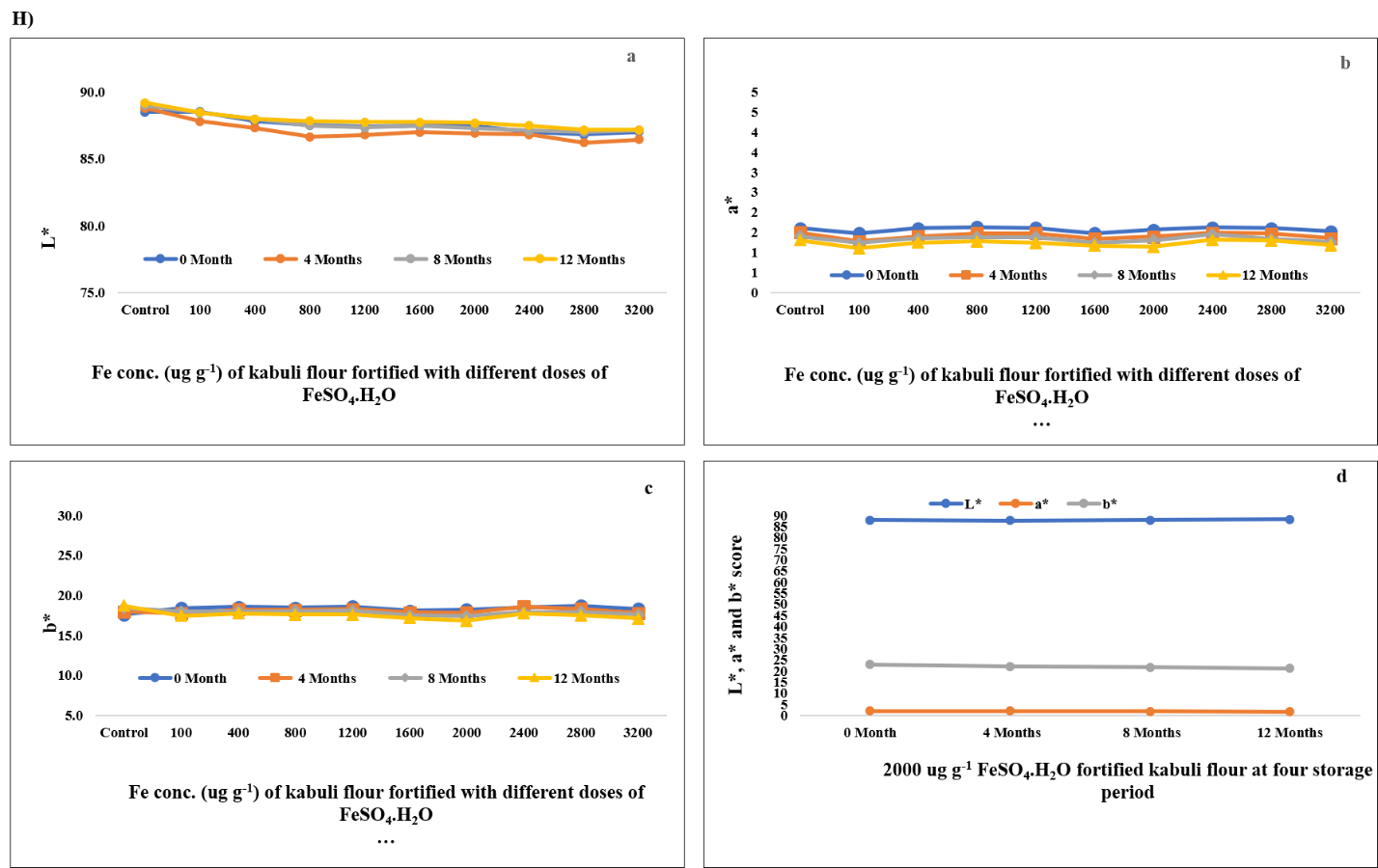


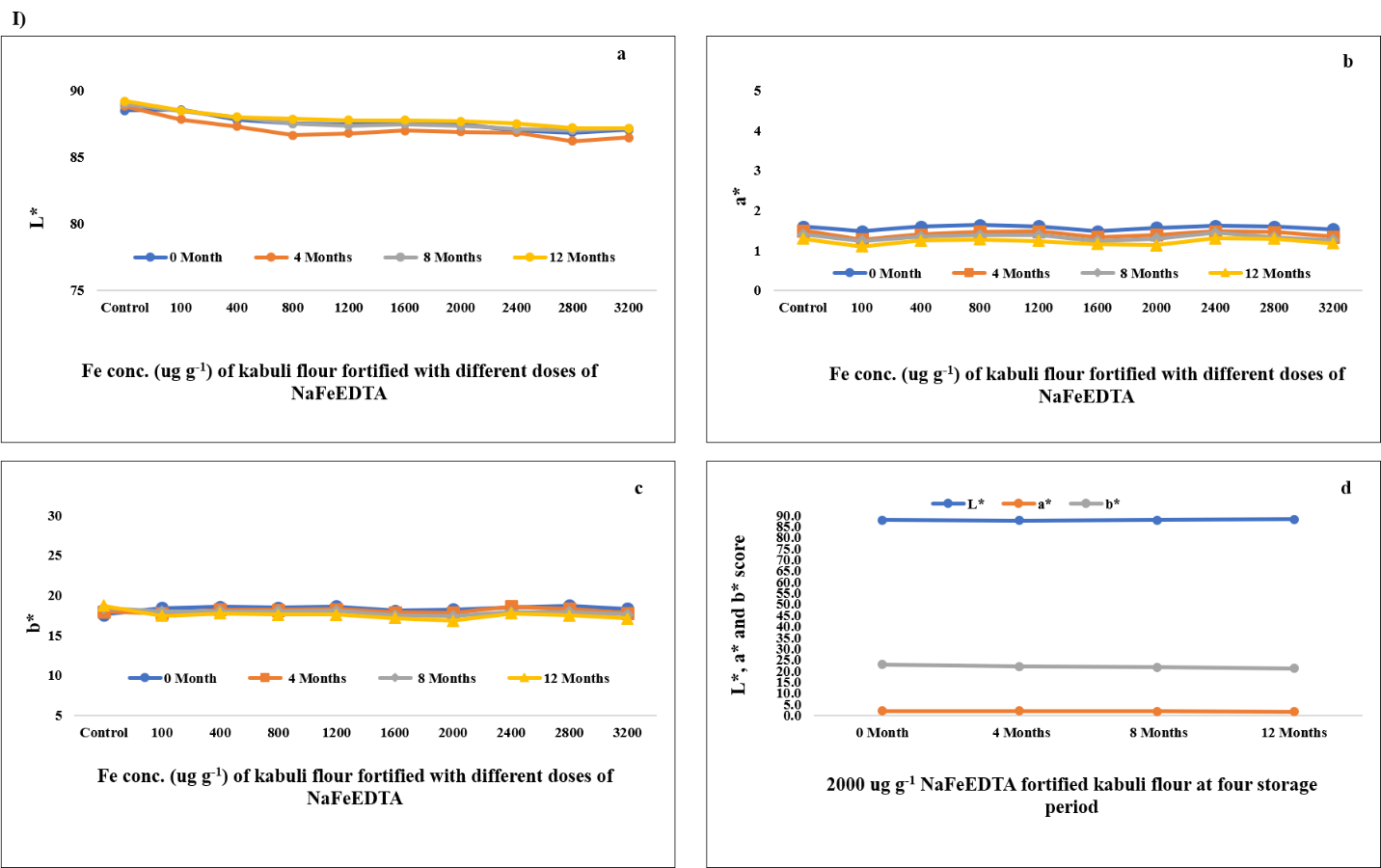


Figure S1. A-I) Represents effect of FeSO_4_.7H_2_O, FeSO_4_.H_2_O, and NaFeEDTA, with nine different doses on color space of split desi seeds, desi flour and kabuli flour samples. Each number shows a) L*(lightness) score b) a*(redness) score c) b*(yellowness) score, and d) L*, a*, b* score at four different storage time at 2000 ug g^-1^ Fe concentration.
