# Supplementary material for: Iron Fortification and Bioavailability of Chickpea (Cicer arietinum L.) Seeds and Flour
Source: Nutrients. 2019 Sep 18;11(9):2240. doi: 10.3390/nu11092240 (PMC6770251; doi:10.3390/nu11092240)
Supplement: Supplementary file 1 [file nutrients-11-02240-s001.zip › Supplementary Figure S2.docx]

Supplementary Figure S2.


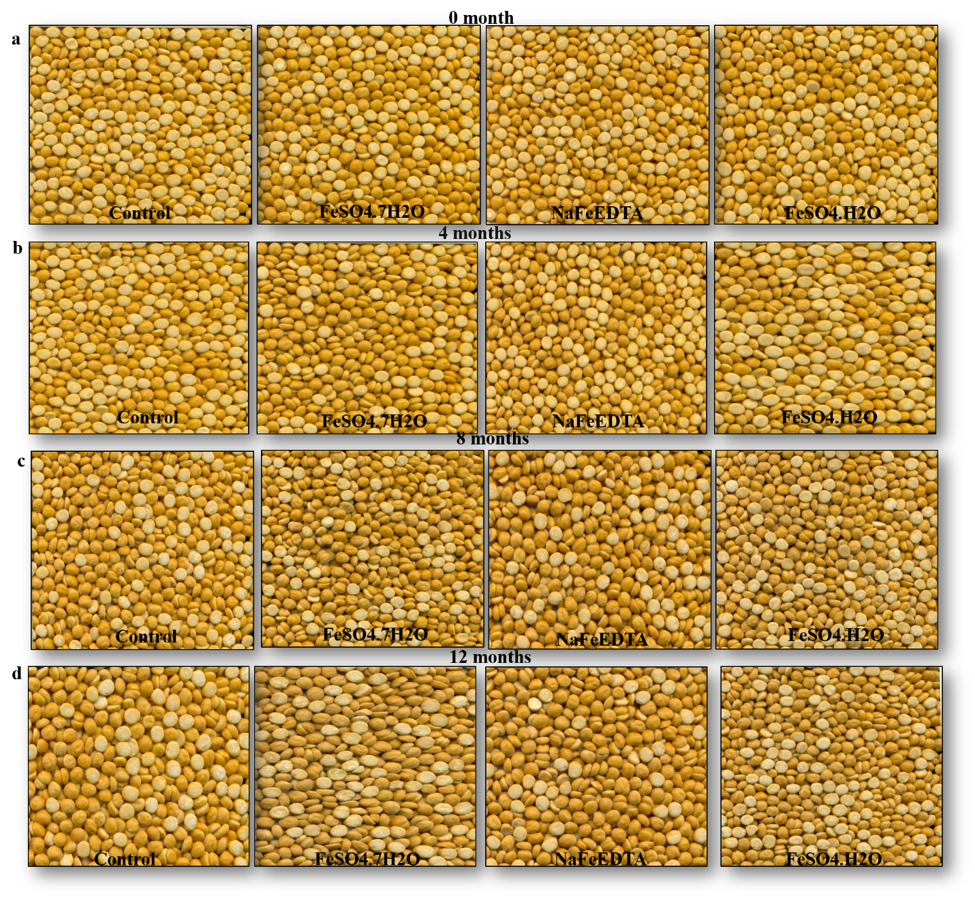


Figure S2. a-d) Images of unfortified (control) and iron-fortified dehulled and split desi seeds with 2000 ug g^-1^ of Fe salt solution prepared from three different Fe salts: FeSO_4_.7H_2_O, NaFeEDTA and FeSO_4_.H_2_O at four different time.
