# Supplementary material for: Iron Fortification and Bioavailability of Chickpea (Cicer arietinum L.) Seeds and Flour
Source: Nutrients. 2019 Sep 18;11(9):2240. doi: 10.3390/nu11092240 (PMC6770251; doi:10.3390/nu11092240)
Supplement: Supplementary file 1 [file nutrients-11-02240-s001.zip › Supplementary Figure S5.docx]

Supplementary Figure S5


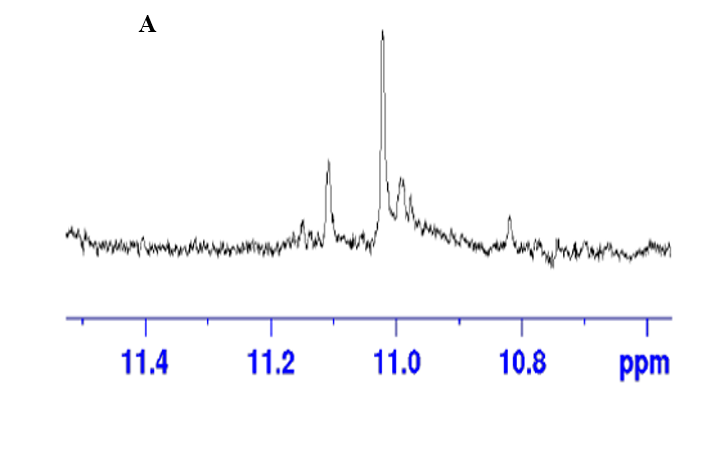


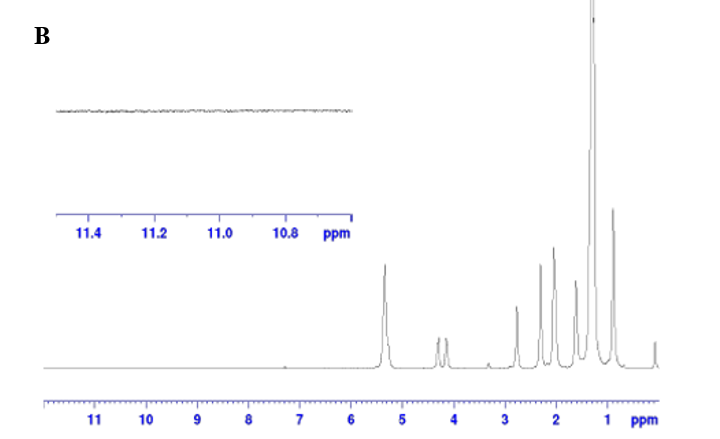


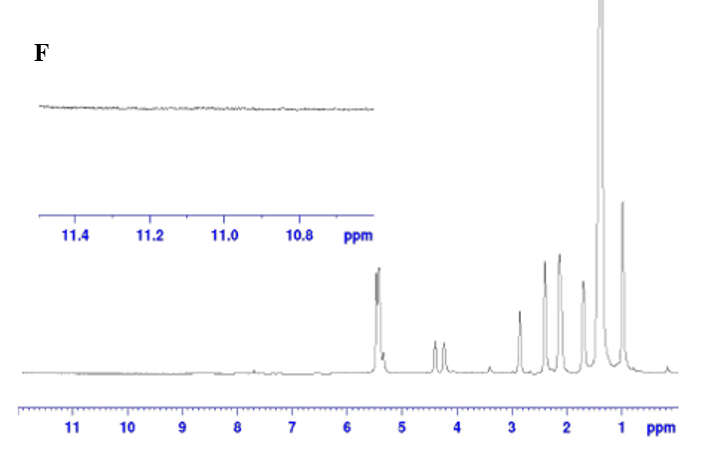

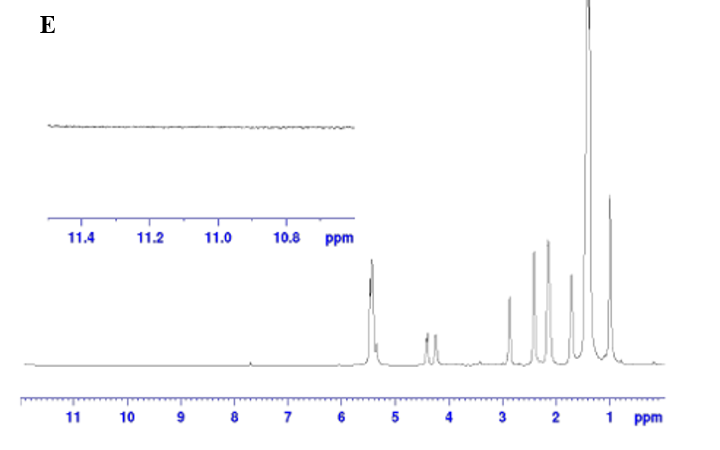

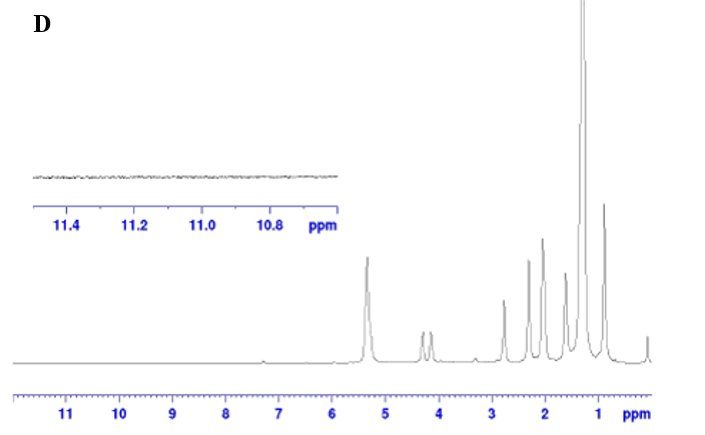

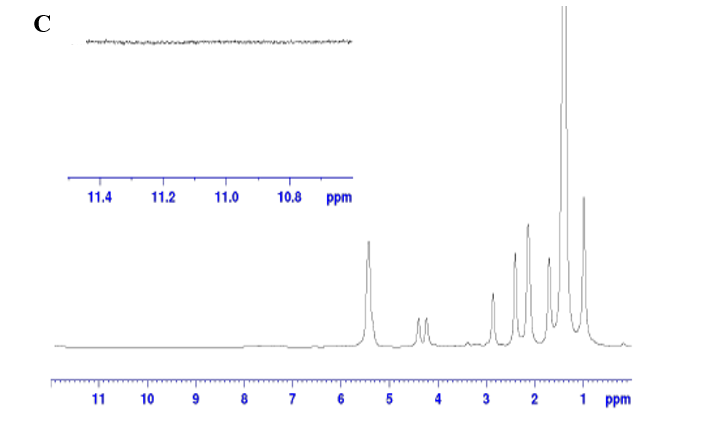


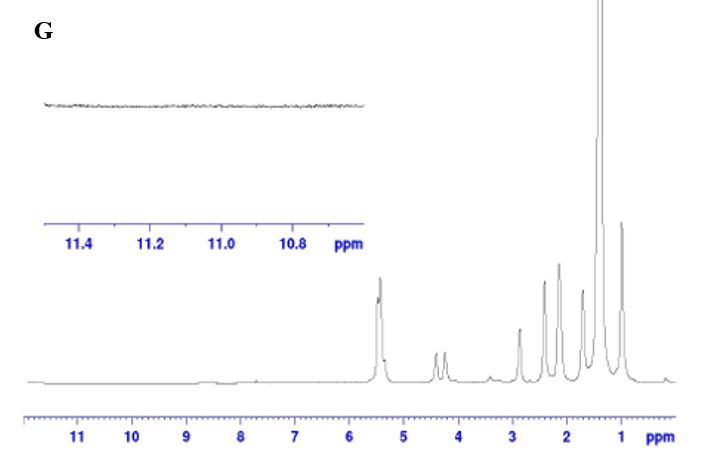

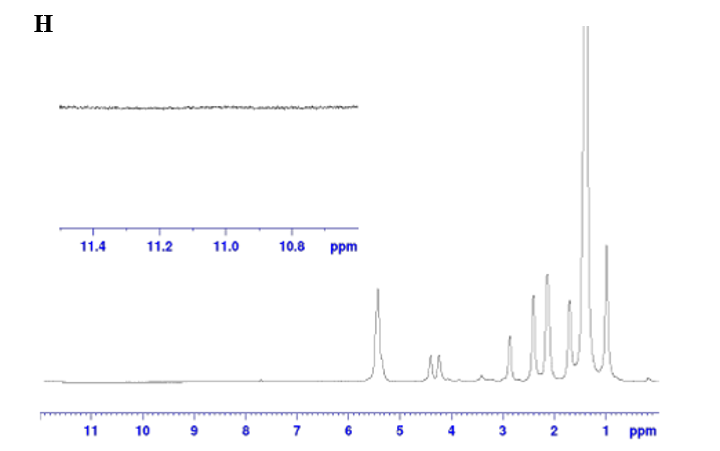


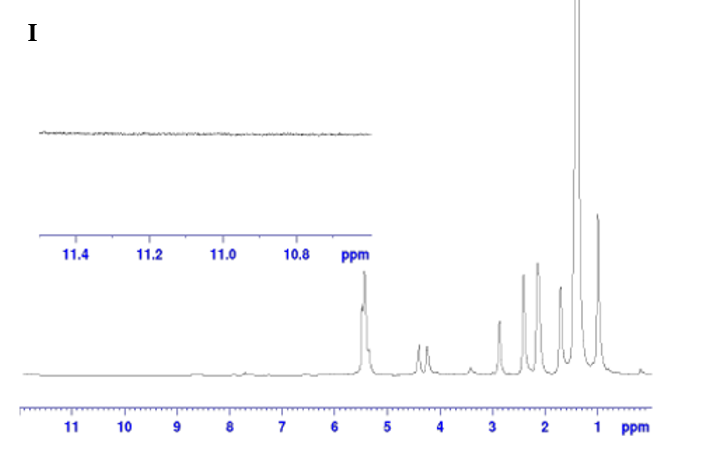
Figure S5. A) Representative sample flaxseed oil shows the hydroperoxide signals in the range of 10.1-11.0 ppm. B-I) After 12 months of storage of desi flour control and desi flour fortified with FeSO4.7H20, NaFeEDTA, and FeSO4.H20 and kabuli flour control and kabuli flour fortified with FeSO4.7H20, NaFeEDTA and FeSO4.H20 with 2000 ug g^-1^ respectively shows no H2O2 signals in the range of 10.1-11.0 ppm.
