# Supplementary material for: Iron Fortification and Bioavailability of Chickpea (Cicer arietinum L.) Seeds and Flour
Source: Nutrients. 2019 Sep 18;11(9):2240. doi: 10.3390/nu11092240 (PMC6770251; doi:10.3390/nu11092240)
Supplement: Supplementary file 1 [file nutrients-11-02240-s001.zip › Supplementary Table S1.docx]

Supplementary Table S1. Analysis of variance of the main effects of three different food matrices [cooked split desi seed (soup), desi chapatti and kabuli chapatti] and three food grade Fe fortificants (FeSO_4_·7H_2_O, FeSO_4_·H_2_O and NaFeEDTA) on Fe bioavailability.

| Source | Food matrices | Fortificants |
| --- | --- | --- |
| DF | 2 | 3 |
| Fe Bioavailability | 0.0011^*^ | 0.1215^ns^ |

DF=Degrees of freedom, ns= non-significant, * = significant at P < 0.05 respectively
